# Automated voxel-based prostate volumetry improves PSA density–based detection of clinically significant prostate cancer in the PSA gray zone: comparison with manual segmentation and the ellipsoid formula

**DOI:** 10.3389/fdgth.2026.1897681

**Published:** 2026-10-07

**Authors:** Jing Han, Yi Qiu, Houdong Liu, Jing Wen

**Affiliations:** 1 Department of Radiology, Suzhou Hospital, Affiliated Hospital of Medical School, Nanjing University, Suzhou, China; 2 Suzhou Research Center of Medical School, Suzhou Hospital, Affiliated Hospital of Medical School, Nanjing University, Suzhou, China; 3 Department of Medical Imaging, Jiangsu Medical College, Yancheng, China

**Keywords:** diagnossis, nnUNet, prostate cancer, PSA density, segmentation

## Abstract

**Purpose:**

To investigate whether voxel-based prostate volume can improve the diagnostic performance of prostate-specific antigen density (PSAD) for detecting clinically significant prostate cancer (csPCa) in patients within the prostate-specific antigen (PSA) gray zone (4–10 ng/mL), and to compare it with conventional formula-based estimation.

**Methods:**

In this retrospective study, 489 patients with PSA levels of 4–10 ng/mL who underwent prostate MRI were included. Prostate volumes were calculated using three methods ellipsoid formula (*PV_formula_*), manual voxel-based segmentation (*PV_mask_*), and automated voxel-based segmentation (*PV_auto_seg_*) based on nnU-Net. Agreement between volumetric measurements was assessed using Bland–Altman analysis, and PSAD was calculated according to three prostate volume estimations. Diagnostic performance for the detection of csPCa was evaluated using receiver operating characteristic (ROC) analysis.

**Results:**

*PV_formula_* systematically overestimated prostate volume compared with *PV_mask_*, with mean volumes of 58.8 ± 28.1 mL and 54.5 ± 26.6 mL (bias 4.4 mL); whereas *PV_auto_seg_* (55.8 ± 20.8 mL) showed close agreement with *PV_mask_* and reduced bias of 1.4 mL. In ROC analysis, voxel-based *PSAD_mask_* (AUC 0.762, 95% CI 0.717–0.808) outperformed the formula-based *PSAD_formula_* (AUC 0.719, 95% CI 0.671–0.767; *P* = 0.01). *PSAD_auto_seg_* calculated from *PV_auto_seg_* yielded an AUC similar (0.754, 95% CI 0.707–0.800) to that of with *PSAD_mask_*, and also outperformed *PSAD_formula_* (*P* = 0.02).

**Conclusion:**

Voxel-based prostate volumetry provides more accurate and consistent volume estimation than the conventional ellipsoid formula, leading to improved PSAD performance for detecting csPCa in the PSA gray zone. Moreover, automated segmentation enables reliable and scalable PSAD calculation with diagnostic performance comparable to manual delineation, supporting its potential for clinical implementation in risk stratification of PCa.

## Introduction

Prostate cancer (PCa) is one of the most frequently diagnosed malignancies in men worldwide and remains a major cause of cancer-related morbidity and mortality (1). Early detection of PCa is crucial for improving prognosis and guiding timely intervention, and screening with PSA has long served as a primary method (2, 3). However, despite its widespread use, PSA has well-recognized limitations, particularly its lack of specificity. Elevated PSA levels may not only reflect malignant disease but can also be caused by benign prostatic hyperplasia, prostatitis, urinary retention, or other nonmalignant conditions (4, 5). This limitation is particularly relevant in the PSA “gray zone”, typically defined as a serum PSA level of 4–10 ng/mL, because PSA values within this range overlap substantially between patients with csPCa and those with benign prostatic conditions, resulting in limited diagnostic specificity and uncertainty regarding biopsy decisions (3). As a result, clinical decision-making in this subgroup remains challenging, often leading either to unnecessary biopsies and overdiagnosis or to delayed detection of clinically relevant tumors (6).

To improve the specificity of PSA, PSAD has been introduced as an important risk stratification parameter, which can partially correct for PSA elevation caused by benign prostate enlargement and has been shown to provide better discrimination of csPCa than PSA alone (7–10). PSAD has been increasingly incorporated into clinical practice and is recognized in several diagnostic pathways and guideline recommendations (11–14). PSAD is generally interpreted together with MRI findings rather than used alone. Recent studies have shown that combining PSAD with PI-RADS can improve csPCa risk stratification and guide biopsy decisions, particularly in patients with negative or equivocal MRI findings. Schoots and Padhani demonstrated progressively increasing csPCa risk across combined PI-RADS and PSAD categories, while Hamidi et al. reported a csPCa detection rate of only 3% in patients with PI-RADS 1–3 findings and PSAD <0.165 ng/mL/mL (15, 16). However, the reliability of PSAD-based risk stratification depends fundamentally on accurate prostate volume measurement. In routine practice, prostate volume is most commonly estimated using the ellipsoid formula based on linear measurements obtained from MRI (17–19). Although this estimation method is simple and convenient, it relies on an idealized geometric assumption that may not accurately reflect the true shape of the prostate, especially in glands with asymmetric enlargement or irregular contours (20, 21). Consequently, formula-based estimation may introduce systematic error, which can directly influence PSAD calculation and potentially weaken its diagnostic performance (22). In contrast, voxel-based volumetric methods derived from MRI allow a more anatomically faithful three-dimensional assessment of the prostate and are generally considered more accurate than conventional formula-based estimation (23).

Recent advances in artificial intelligence have further expanded the feasibility of precise prostate volumetry in clinical practice (24). Deep learning–based automated segmentation methods, particularly nnU-Net, have shown strong generalizability across biomedical segmentation tasks, while studies in prostate MRI have demonstrated that deep learning algorithms can achieve prostate volume assessment comparable to radiologists (25). These developments provide an opportunity to integrate accurate, reproducible, and scalable volumetric assessment into routine clinical workflows without substantially increasing reader burden (26). However, despite growing interest in prostate segmentation and automated imaging analysis, the extent to which different prostate volume estimation methods influence PSAD performance for detecting csPCa remains insufficiently explored, especially in patients within the PSA gray zone. Therefore, the aim of this study was to compare formula-based and voxel-based prostate volume estimation methods, evaluate the agreement between manual and automated volumetric measurements, and investigate how these different approaches affect the diagnostic performance of PSAD for detecting clinically significant prostate cancer in patients with PSA levels of 4–10 ng/mL.

## Methods

### Study design and clinical cohort

This retrospective study was approved by the institutional review board (IRB), which waived the requirement for written informed consent, and was conducted in accordance with the Declaration of Helsinki. Consecutive patients who underwent prostate MRI followed by systematic transrectal ultrasound–guided prostate biopsy and/or MRI-transrectal ultrasound fusion–targeted biopsy between January 2022 and October 2025 were screened. The inclusion criteria were as follows: 1) serum PSA level of 4–10 ng/mL; 2) availability of prebiopsy prostate MRI including axial T2-weighted imaging; and 3) histopathological confirmation by systematic biopsy, targeted biopsy, or radical prostatectomy. The exclusion criteria were: 1) previous prostate surgery or treatment; 2) inadequate MRI quality because of artifacts; and 3) incomplete clinical or imaging data. Clinically significant prostate cancer was defined as International Society of Urological Pathology grade group ≥2 (Gleason score ≥3 + 4). All patients underwent systematic biopsy, and MRI-transrectal ultrasound fusion-targeted biopsy was additionally performed for lesions with a PI-RADS v2.1 score ≥3. The patient selection process is presented in Figure 1.

**Figure 1:**
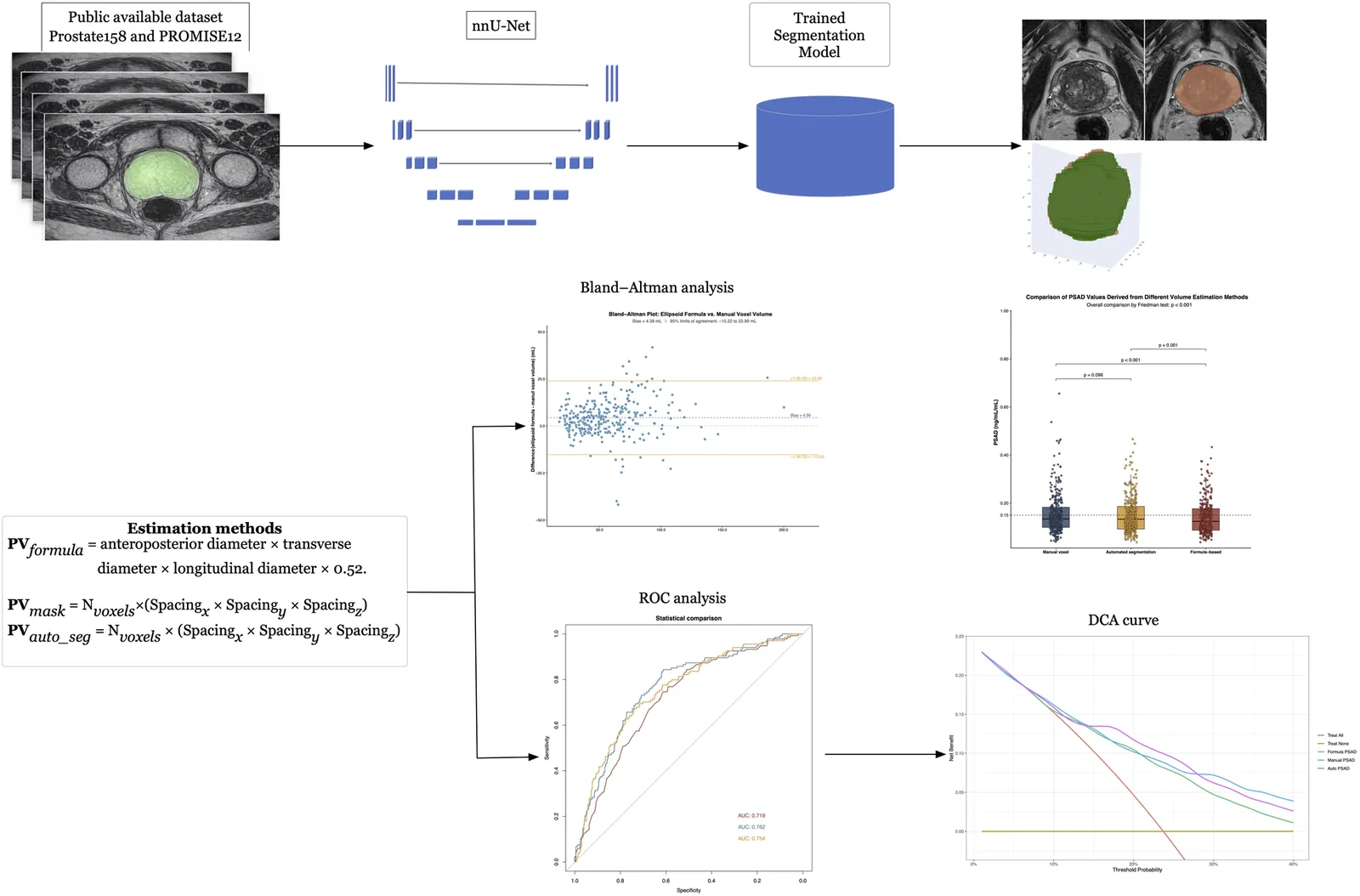
Workflow of this study.

### Datasets for automated segmentation model development and validation

The automated segmentation model was developed exclusively using the PROSTATE158 dataset, an expert-annotated dataset comprising 158 biparametric prostate MRI examinations acquired at 3.0 T (27). External technical validation was performed using the 50 publicly available labeled cases from the PROMISE12 dataset (28). The in-house clinical cohort of 489 patients was entirely independent of model development and was not used for training, validation, hyperparameter tuning, model selection, or postprocessing. After model development and technical validation had been completed, the finalized model was applied to the in-house cohort without retraining or dataset-specific adaptation.

### MRI acquisition

All prostate MRI examinations were performed using two 3.0-T MRI scanners (MAGNETOM Skyra, Siemens AG) with a 32-channel phased-array pelvic coil. In accordance with PI-RADS v2.1 recommendations, the imaging protocol included axial turbo spin-echo (TSE) T2WI, sagittal TSE T2WI, and axial diffusion-weighted imaging (DWI). The main acquisition parameters were as follows: axial T2WI, repetition time (TR) = 4400 ms, echo time (TE) = 96 ms, slice thickness=3.0 mm, field of view (FOV) = 230 × 230 mm, matrix=320 × 224; sagittal T2WI, TR = 3800 ms, TE = 117 ms, slice thickness=3.0 mm, FOV=230 × 230 mm, matrix=320 × 256; and axial DWI, TR = 6570 ms, TE = 88 ms, slice thickness=3.0 mm, FOV=320 × 224 mm, matrix=192 × 192, with multiple b values of 0, 100, and 1000 s/mm^2^.

### Manual prostate segmentation and volume measurement

For each of the 489 in-house cases, whole-gland prostate segmentation was independently performed on axial T2-weighted images by two radiologists using 3D Slicer software (version 5.0.1). Both radiologists had more than 20 years of experience in prostate MRI and were blinded to the histopathological outcomes and to each other's segmentations. The prostate gland was delineated slice-by-slice to generate a three-dimensional binary mask. The two independently generated masks were subsequently reviewed by a third radiologist with more than 20 years of experience in prostate MRI. Any discrepancies were adjudicated, and the reviewed mask was used as the final manual reference segmentation for volumetric and automated-segmentation performance analyses. According to PI-RADS, the prostate volume was calculated on T2WI using the following ellipsoid formula:

$$PV_{\mathrm{formula}}\;=\;\mathrm{anteroposterior}\;\mathrm{diameter}\;\times \;\mathrm{transverse}\;\mathrm{diameter}\;\times \;\mathrm{longitudinal}\;\mathrm{diameter}\;\times \;0.52$$

The voxel-based prostate volume was calculated as

$$PV_{\mathrm{mask}}\;=\;N_{voxels}\times (\mathrm{Spacingx}\;\times \;\mathrm{Spacingy}\;\times \;\mathrm{Spacingz})$$

where ***N****_voxels_* is the number of voxels within the segmentation mask, and *Spacing_x_*, *Spacing_y_*, and *Spacing_z_* represent voxel spacing in the three orthogonal directions obtained from original DICOM image metadata. Based on different volume estimation methods, PSAD was calculated as follows:

$$PSAD_{\;formula}\;=\;tPSA/PV_{\;formula}$$

$$PSAD_{mask}\;=\;tPSA/\;PV_{mask}$$

### Automated prostate segmentation and volumetry

Automated whole-gland prostate segmentation was performed using the nnU-Net framework on only axial T2WI (25). Model development and validation datasets were used as described above. Images and corresponding reference labels were converted into the standard nnU-Net format. Image resampling, cropping, intensity normalization, network configuration, patch size, batch size, and data augmentation were determined using the self-configuring nnU-Net pipeline. The segmentation task was formulated as a binary segmentation task, with background labeled as 0 and prostate labeled as 1. All nonzero labels were merged into a single prostate class to ensure consistency of whole-gland segmentation. Other hyperparameters, including patch size, batch size, and data augmentation strategy, were automatically configured by the nnU-Net framework. Segmentation performance was evaluated using the Dice similarity coefficient (DSC) and the 95th percentile Hausdorff distance (HD95). For each case, an automated whole-gland segmentation mask was generated, and voxel-based automated prostate volume was calculated using the same formula as for manual segmentation:

$$PV_{auto\_seg}\;=\;N_{voxels}\;\times \;(\mathrm{Spacingx}\;\times \;\mathrm{Spacingy}\;\times \;\mathrm{Spacingz})$$

where ***N****_voxels_* denotes the number of voxels within the automated segmentation mask, the corresponding PSAD was calculated from automated segmentation:

$$PSAD_{auto\_seg}\;=\;tPSA/PV_{auto\_seg}$$

### Statistical analysis

Continuous variables were expressed as mean ± SD or median with interquartile range (IQR), as appropriate, whereas categorical variables were summarized as counts and percentages. Agreement between prostate volume measurements was evaluated using Bland-Altman analysis. The diagnostic performance of prostate volume and PSAD derived from different measurement methods for identifying csPCa was assessed using ROC curve analysis. Four multivariable logistic regression models were constructed. Model 0 included age, serum PSA, and PI-RADS. Formula-derived PSAD, manually derived voxel-based PSAD, and automatically derived voxel-based PSAD were separately added to Model 0 to construct Models 1–3, respectively. Interobserver agreement for manual prostate volume measurements was assessed using intraclass correlation coefficient (ICC), with its 95% confidence interval. ICC values of <0.50, 0.50–0.75, 0.75–0.90, and >0.90 were interpreted as indicating poor, moderate, good, and excellent agreement, respectively. The three PSAD measurements were not entered simultaneously because they represented highly correlated alternative measurements of the same parameter. Model discrimination was quantified using the AUC, and correlated AUCs were compared using the DeLong test. The AUC was calculated for each method and compared using the DeLong test, and optimal cutoff values were determined using the Youden index. Decision curve analysis was performed to evaluate the clinical utility of different PSAD-based approaches across a range of threshold probabilities. All statistical analyses were performed using R software (version 4.2.0), and a two-sided *P* value <0.05 was considered statistically significant.

## Results

### Patient characteristics

A total of 489 patients were included in this study, including 109 patients (22.29%) with csPCa and 380 patients (77.71%) without csPCa. The mean PSA level was 6.59 ± 1.69 ng/mL, and the mean age was 67.5 ± 7.6 years (Table 1). The distribution of PI-RADS scores is summarized in Table 1. For whole-gland segmentation, the nnU-Net model achieved favorable performance, with mean DSCs of 0.907 ± 0.04 in the external validation cohort and 0.894 ± 0.04 in our in-house dataset. The corresponding HD95 were 4.45 ± 2.61 and 4.47 ± 2.63, respectively, indicating good segmentation accuracy (Figure 2). Manual prostate volume measurements demonstrated excellent interobserver agreement between the two independent readers across the 489 cases, with an ICC of 0.96 (95% CI, 0.95–0.97). These findings supported the reproducibility of the reviewed manual segmentations used as the reference standard.

**Table 1:** Clinical characteristics of the study population. Demographic characteristics.

Variable	csPCa (*n* = 109)	Non-csPCa (*n* = 380)	*P*	Overall (*n* = 489)
Age (years, mean ± SD)	69.9 ± 8.3	66.8 ± 7.2	<0.001	67.5 ± 7.6
PSA (ng/mL, mean ± SD)	6.75 ± 1.68	6.54 ± 1.70	0.24	6.59 ± 1.69
Formula-derived PV (mL, median, IQR)	38.8 (27.9–54.7)	61.3 (44.1–73.9)	<0.001	53.1 (38.6–73.9)
Formula-derived PSAD (ng/mL/mL, median, IQR)	0.17 (0.12–0.24)	0.11 (0.08–0.15)	<0.001	0.12 (0.09–0.18)
Gleason Score				
≤3 + 3	380			
3 + 4	43			
4 + 3	28			
4 + 4	22			
>4 + 4	16			
PI-RADS				
2	181			
3	113			
4	143			
5	52			

csPCa, clinically significant prostate cancer; IQR, interquartile range; PSA, prostate-specific antigen; PSAD, prostate-specific antigen density; PV, prostate volume; SD, standard deviation.

**Figure 2:**
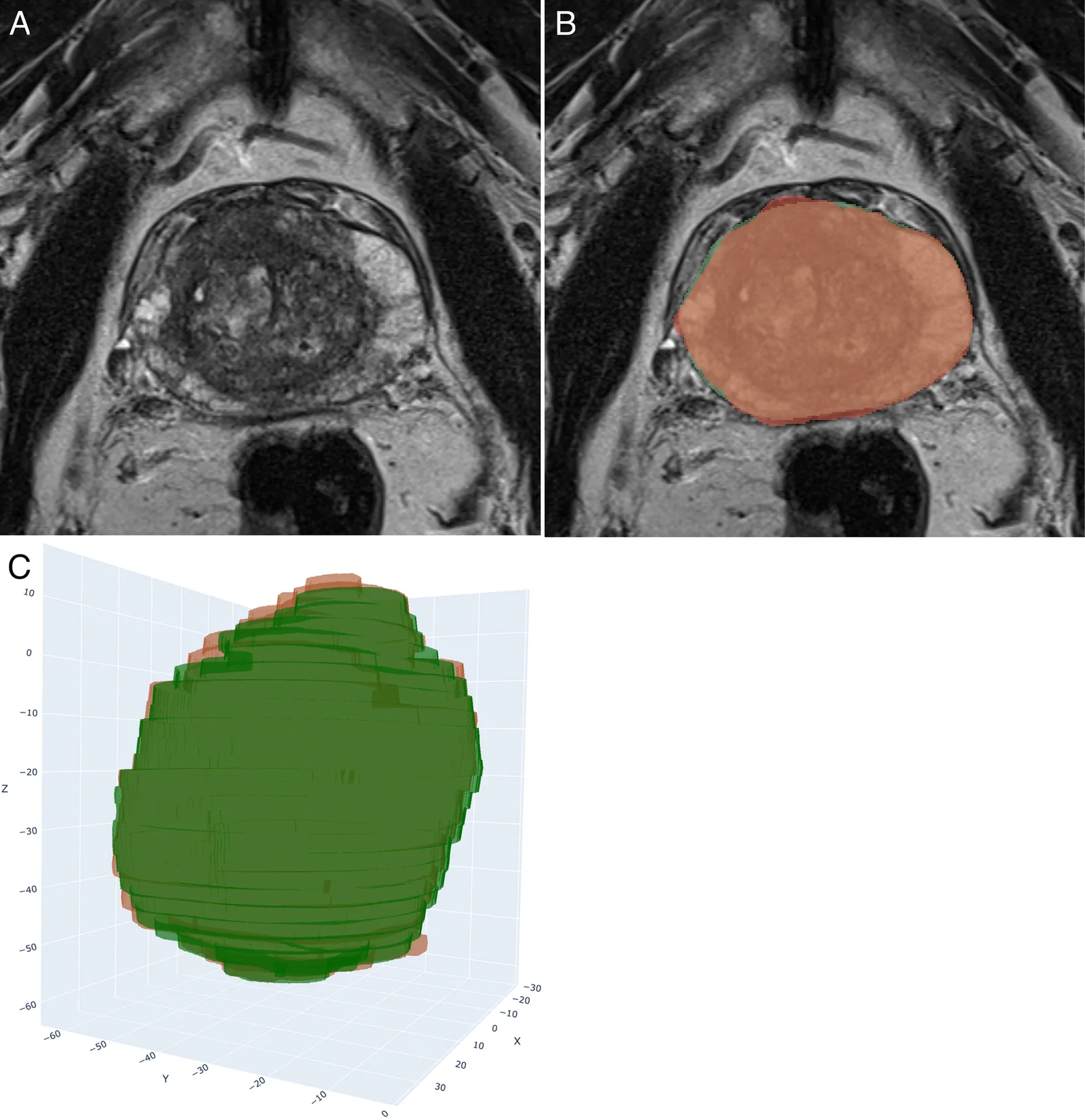
Whole-gland prostate segmentation. (**A**) Original axial T2-weighted image. (**B**) Two-dimensional overlay of the automated nnU-Net segmentation (red) and manual reference segmentation delineated by expert radiologists (green). (**C**) Three-dimensional rendering of the automated segmentation (red) and manual reference segmentation (green).

### Prostate volume and PSAD derived from different measurement methods

The median prostate volumes were 53.1 mL (IQR 38.6–73.8) for *PV_formula_*, 49.6 mL (IQR 35.8–67.4) for *PV_mask_*, and 50.4 mL (IQR 36.0–70.9) for *PV_auto_seg_*. Accordingly, the calculated PSAD values were 0.124 ng/mL/mL (IQR 0.087–0.178) for *PSAD_formula_*, 0.133 ng/mL/mL (IQR 0.092–0.192) for *PSAD_mask_*, and 0.131 ng/mL/mL (IQR 0.098–0.184) for *PSAD_auto_seg_*.

### Agreement among different prostate volume measurement methods

As shown in Figure 3A, Bland-Altman analysis demonstrated that the mean difference (bias) between *PV_formula_* and *PV_mask_* was 4.4 mL, with 95% limits of agreement ranging from −15.2 to 24.0 mL. The bias between *PV_formula_* and *PV_auto_seg_* was 3.0 mL, with 95% limits of agreement from −17.5 to 23.6 mL (Figure 3B). In contrast, the agreement between *PV_auto_seg_* and *PV_mask_* was substantially closer, with a smaller bias of 1.4 mL and narrower 95% limits of agreement ranging from −9.9 to 12.6 mL, indicating that automated segmentation provided higher precision and close agreement with manual mask-based volumetry (Figure 3C). Figure 4 further illustrates the median, 25th, and 75th percentile values of the three volume measurements.

**Figure 3:**
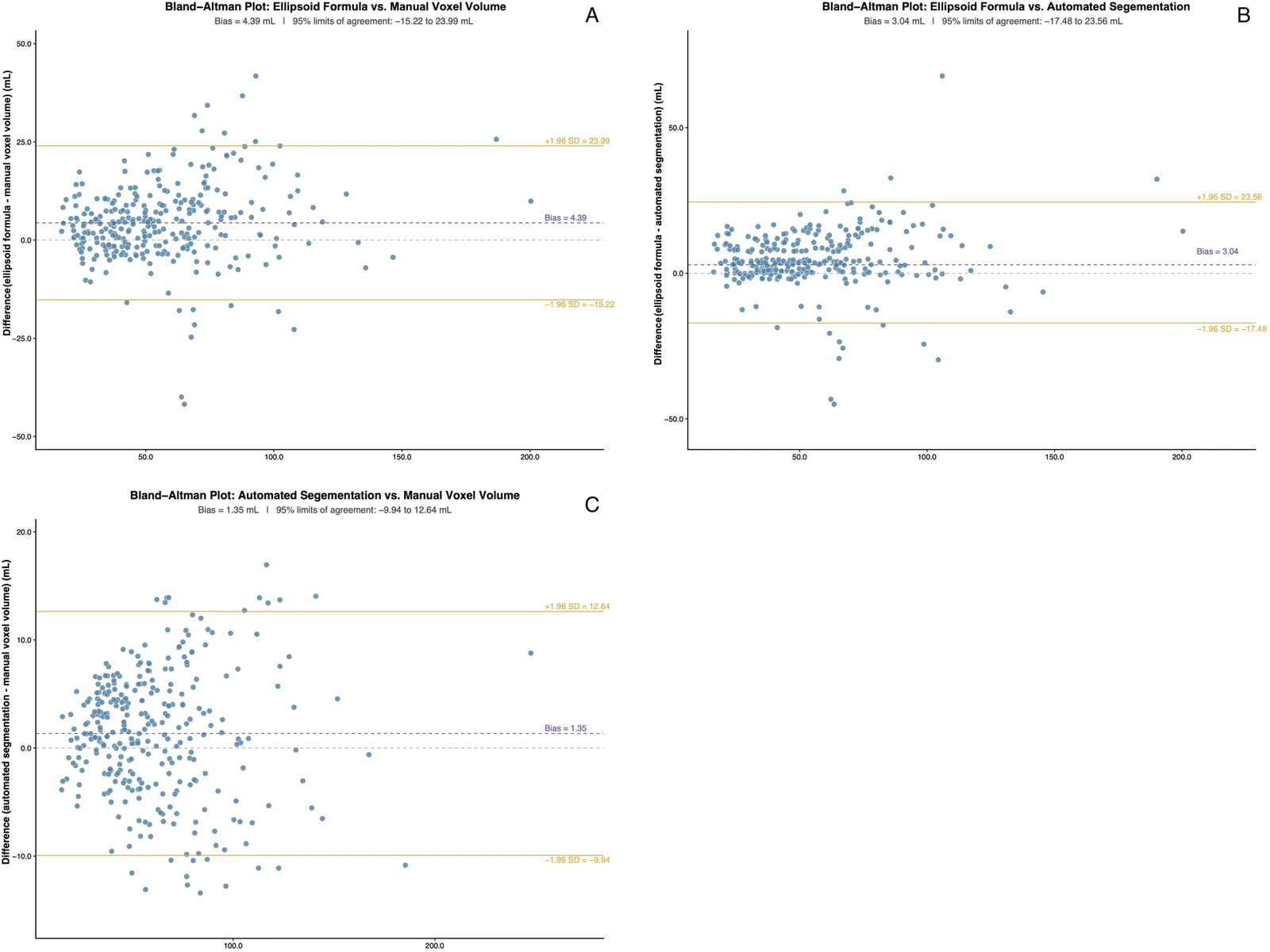
Bland–altman plots comparing the three prostate volume estimation methods. The purple dashed line represents the mean bias, the orange solid lines represent the 95% limits of agreement calculated as the mean bias ± 1.96 SD of the paired differences, and the gray dashed line represents zero difference. **A**, ellipsoid formula minus manual voxel volume; **B**, ellipsoid formula minus automated segmentation volume; **C**, automated segmentation volume minus manual voxel volume.

**Figure 4:**
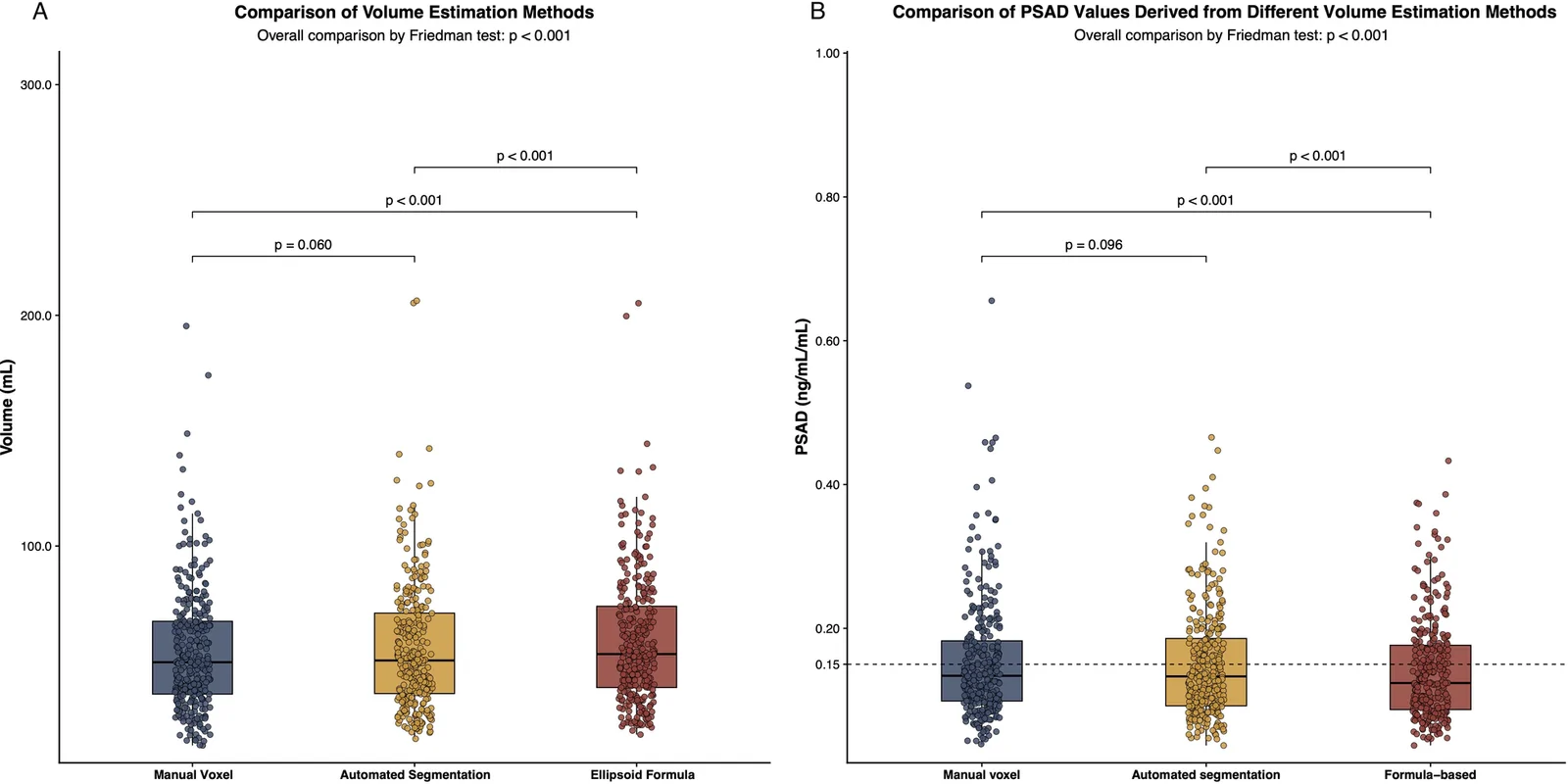
Distributions of **(A)** prostate volume and **(B)** PSA density derived from the ellipsoid formula, manual voxel-based segmentation, and automated segmentation. The dashed horizontal line in panel **B** indicates a PSAD threshold of 0.15 ng/mL/mL.

### Diagnostic performance of prostate volume and PSAD for detecting csPCa

The diagnostic performance of various prostate volume and PSAD methods for identifying csPCa is summarized in Figure 5. For prostate volume alone, both *PV_mask_* (AUC 0.746, 95% CI 0.686–0.806) and *PV_auto_seg_* (AUC 0.739, 95% CI 0.678–0.801) demonstrated significantly superior diagnostic accuracy compared to *PV_formula_* (AUC 0.708, 95% CI 0.645–0.772; *P* = 0.02 and *P* = 0.03, respectively). Notably, no significant difference was observed between *PV_mask_* and *PV_auto_seg_* (*P* = 0.21). Similarly, PSAD derived from voxel-based prostate volume measurements exhibited better diagnostic performance than formula-based PSAD. Specifically, the AUCs were 0.719 (95% CI 0.671–0.767) for *PSAD_formula_*, 0.762 (95% CI 0.717–0.808) for *PSAD_mask_*, and 0.754 (95% CI 0.707–0.800) for *PSAD_auto_seg_*. Furthermore, *PSAD_mask_* and *PSAD_auto_seg_* significantly outperformed *PSAD_formula_* (*P* = 0.01 and *P* = 0.02, respectively), while no significant difference was found between *PSAD_mask_* and *PSAD_auto_seg_* (*P* = 0.57). Decision curve analysis showed that voxel-based PSAD generally provided equal or greater net benefit than formula-based PSAD across most clinically relevant threshold probabilities (Figure 6). At the Youden-optimal cutoffs of 0.159, 0.170, and 0.168, formula-derived, manual voxel-based, and automated PSAD achieved sensitivities of 59.6%, 65.1%, and 64.2% and specificities of 75.8%, 77.1%, and 76.6%, respectively. The corresponding missed-csPCa rates were 40.4%, 34.9%, and 35.8%, while the potential biopsy avoidance rates were 67.9%, 67.7%, and 67.5% (Table 2).

**Figure 5:**
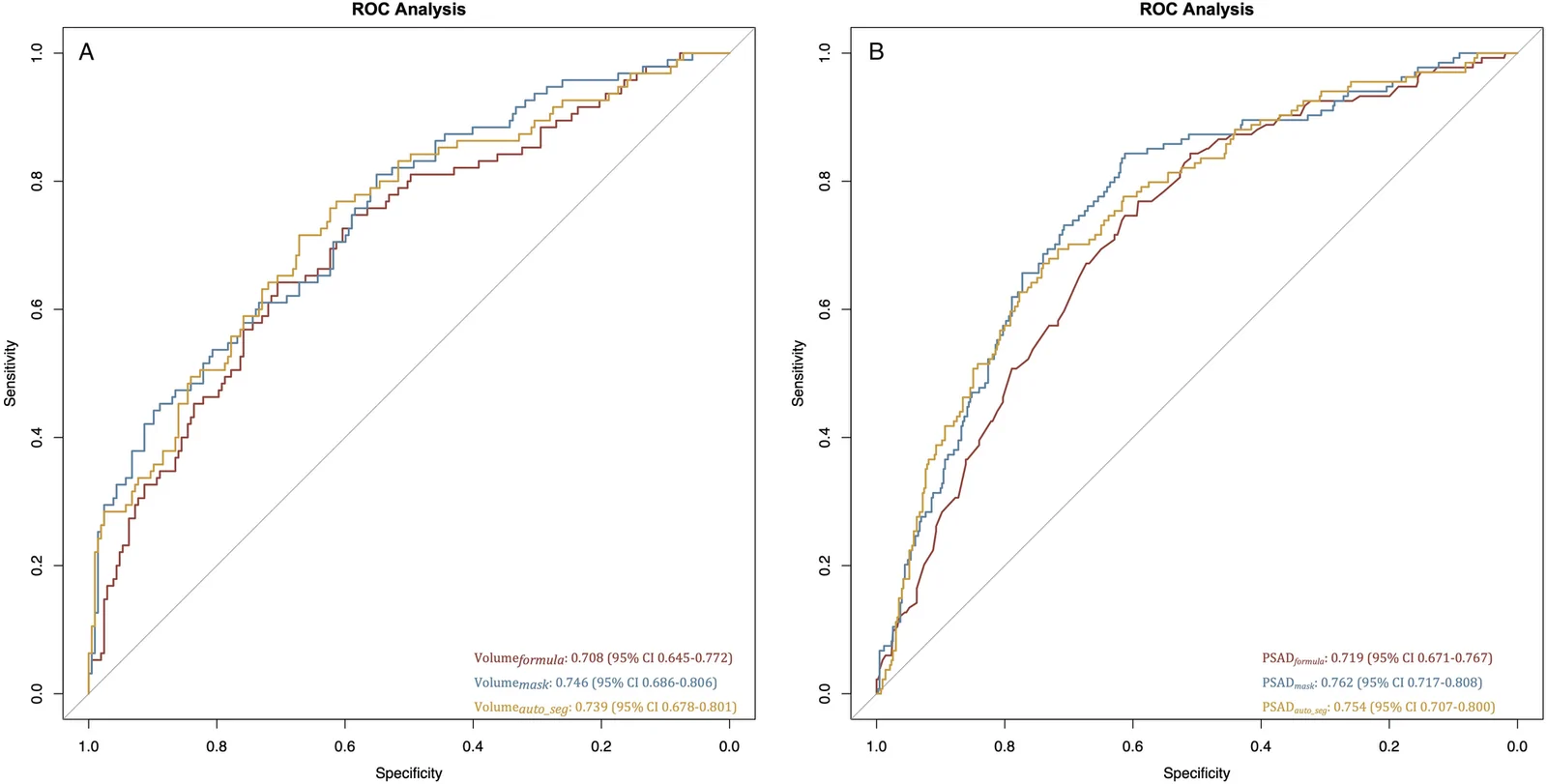
ROC curves for detecting clinically significant prostate cancer using prostate volume and PSAD derived from the three measurement methods. **A**, prostate volume; **B**, PSAD.

**Figure 6:**
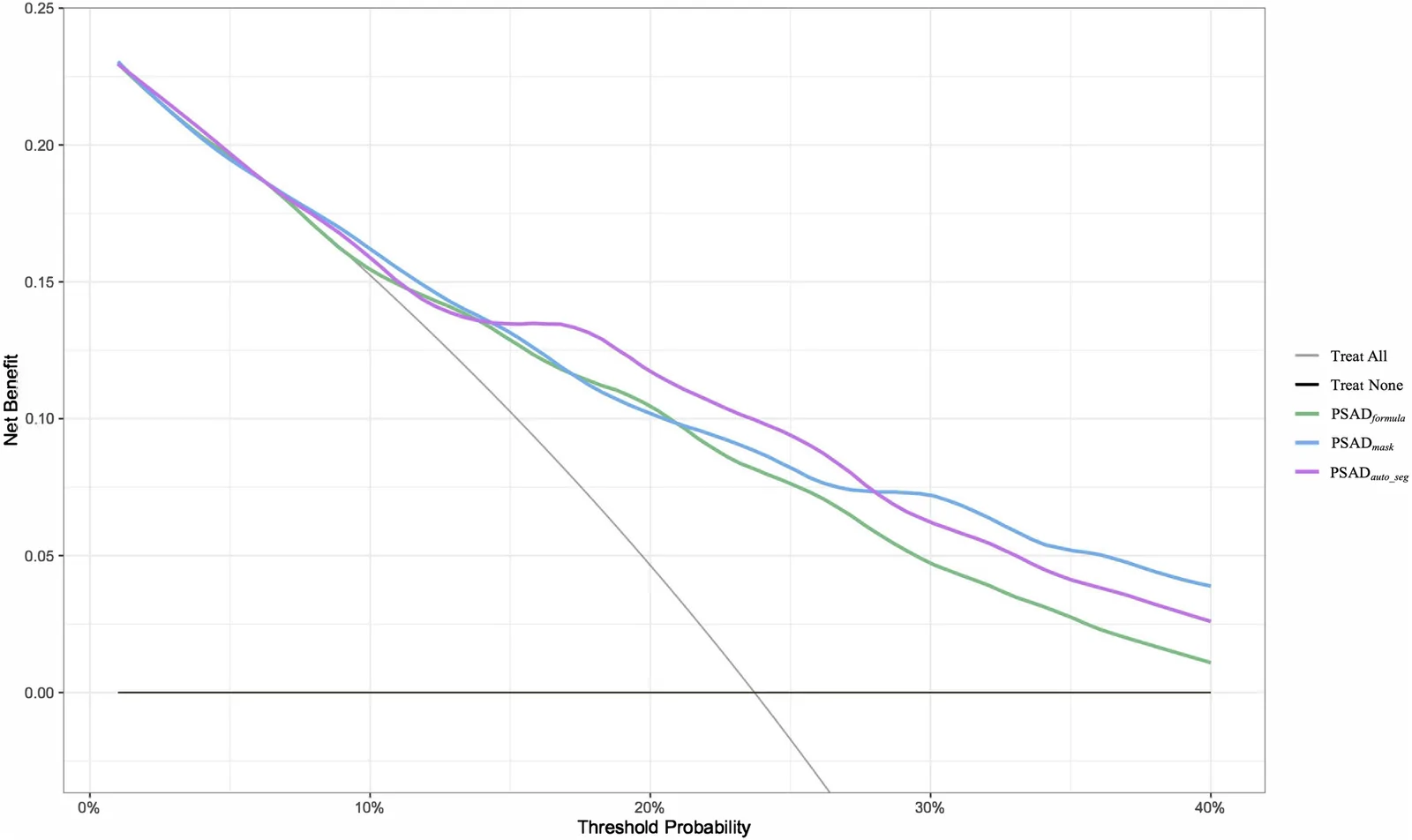
Decision curve analysis of PSAD derived from the three prostate volume measurement methods.

**Table 2:** Diagnostic performance and biopsy-related outcomes of different PSAD methods for detecting csPCa.

PSAD method	Cutoff (ng/mL/mL)	Sensitivity	Specificity	PPV	NPV	Accuracy	Missed csPCa	csPCa among avoided biopsies	Potential biopsy avoidance
Formula-derived	0.159	59.6%	75.8%	41.4%	86.7%	72.2%	44/109 (40.4%)	44/332 (13.3%)	332/489 (67.9%)
Manual voxel-based	0.170	65.1%	77.1%	44.9%	88.5%	74.4%	38/109 (34.9%)	38/331 (11.5%)	331/489 (67.7%)
Automated voxel-based	0.168	64.2%	76.6%	44.0%	88.2%	73.8%	39/109 (35.8%)	39/330 (11.8%)	330/489 (67.5%)

Optimal cutoffs were determined by maximizing the Youden index. A biopsy was considered avoided when the corresponding PSAD value was below the cutoff.

csPCa, clinically significant prostate cancer; NPV, negative predictive value; PPV, positive predictive value; PSAD, prostate-specific antigen density.

### Incremental value of voxel-based PSAD

The baseline model comprising age, serum PSA, and PI-RADS yielded an AUC of 0.878 (95% CI, 0.838–0.919). The addition of formula-derived PSAD increased the AUC to 0.905 (95% CI, 0.868–0.942; *Δ*AUC = 0.027; *P* = 0.001), as demonstrated in Table 3. The addition of manually derived voxel-based PSAD and automatically derived voxel-based PSAD further increased the AUC to 0.916 (95% CI, 0.881–0.950; *Δ*AUC = 0.038; *P* < 0.001) and 0.910 (95% CI, 0.873–0.947; *Δ*AUC = 0.032; *P* < 0.001), respectively. Compared with the formula-derived PSAD model, the manually derived voxel-based PSAD model showed a significantly higher AUC (*P* = 0.048), whereas the improvement observed for the automatically derived voxel-based PSAD model did not reach statistical significance (*P* = 0.152).

**Table 3:** Incremental diagnostic performance of models incorporating different PSAD measures beyond age, PSA, and PI-RADS for detecting csPCa.

Model	Predictors	AUC (95% CI)	*P* (vs. Model 0)	*P* (vs. Model 1)
Model 0	Age + PSA + PI-RADS	0.878 (0.838–0.919)	Reference	/
Model 1	Model 0 + PSAD*_formula_*	0.905 (0.868–0.942)	0.001	Reference
Model 2	Model 0 + PSAD*_mask_*	0.916 (0.881–0.950)	<0.001	0.048
Model 3	Model 0 + PSAD*_auto_seg_*	0.910 (0.873–0.947)	<0.001	0.152

AUC, area under the receiver operating characteristic curve; csPCa, clinically significant prostate cancer; PSAD, prostate-specific antigen density.

### PI-RADS 3 subgroup analysis

Among the 113 patients with PI-RADS 3 findings, 21 had csPCa and 92 did not. At the prespecified whole-cohort optimal cutoffs, formula-derived PSAD yielded a sensitivity of 57.1%, specificity of 78.3%, PPV of 37.5%, and NPV of 88.9% (Supplementary Table S1). Manual voxel-based PSAD yielded a sensitivity of 71.4%, specificity of 71.7%, PPV of 36.6%, and NPV of 91.7%, whereas automated voxel-based PSAD yielded corresponding values of 66.7%, 73.9%, 36.8%, and 90.7%, respectively. Compared with formula-derived PSAD, manual and automated voxel-based PSAD correctly classified three and two additional csPCa cases as positive, respectively, but potentially avoided nine and six fewer biopsies.

## Discussion

In this study, we evaluated the impact of different prostate volume estimation methods and corresponding PSAD values, as well as their diagnostic performance for the detection of csPCa in patients within the PSA gray zone (serum PSA level of 4–10 ng/mL). Our analyses demonstrated that formula-based prostate volume estimation systematically yielded larger prostate volumes than manual delineation (bias 4.39 mL) and automated segmentation (bias 3.04 mL) voxel-based measurement, while the latter two estimation methods demonstrated close agreement (bias 1.35 mL). Moreover, both voxel-based approaches improved the diagnostic performance of prostate volume and calculated PSAD values for identifying csPCa as compared with the conventional ellipsoid formula, whereas automated and manual voxel-based methods showed comparable performance. In addition to improved discrimination, voxel-based PSAD approaches also provided greater net benefit than formula-based PSAD on decision curve analysis, suggesting superior clinical utility for biopsy decision-making in patients within the PSA gray zone.

Previous studies have consistently shown that PSAD improves risk stratification beyond PSA alone, particularly when interpreted together with MRI findings or PI-RADS assessment (7, 11, 12). Schoots et al. proposed a risk-adapted biopsy strategy based on combined PI-RADS and PSAD categories, demonstrating that PSAD substantially modifies csPCa risk in patients with negative or equivocal MRI findings (16). Similarly, Hamidi et al. reported that combining PI-RADS with a PSAD cutoff of 0.165 ng/mL/mL identified patient groups with markedly different csPCa detection rates and could potentially reduce unnecessary biopsies (15). However, prostate volume in these and most previous clinical studies was estimated using the conventional ellipsoid formula, and the potential influence of volume measurement error on PSAD-based risk classification was not specifically addressed (20, 22). The present study extends this evidence by demonstrating that the method used to estimate prostate volume affects both PSAD values and their diagnostic performance, with manual and automated voxel-based volumetry providing better discrimination of csPCa than formula-based volumetry.

The PSA gray zone remains a major clinical challenge because PSA values in this range are frequently influenced by benign conditions, reducing the specificity of PSA for prostate cancer detection (29). In this context, PSAD has been widely used as a more refined biomarker because it incorporates prostate volume into PSA interpretation (30). However, the clinical value of PSAD depends directly on the accuracy of prostate volume measurement (12). Our results support this concept by showing that formula-based volume estimation produced larger median prostate volumes than both mask-based and automated voxel-based measurements. As PSAD is calculated by dividing PSA by prostate volume, overestimation of prostate volume inevitably lowers PSAD values and may weaken its ability to identify patients with csPCa (22, 31). This mechanism likely explains why PSAD derived from formula-based volume showed inferior diagnostic performance compared with PSAD based on voxel-derived measurements.

The Bland–Altman analysis further clarified the relationship among the three volume estimation methods. Although formula-based volume showed a positive bias compared with both mask-based and automated volumetry, the agreement between automated segmentation and manual mask-based measurement was substantially closer, with a smaller mean difference and narrower limits of agreement. These findings indicate that automated segmentation can reproduce manual volumetric assessment with relatively higher precision. This is also supported by the segmentation performance metrics, with Dice similarity coefficients of 0.907 in the external validation cohort and 0.894 in our dataset, alongside low HD95 values. Taken together, these results suggest that the automated model provided robust whole-gland segmentation and a reliable basis for downstream volume and PSAD calculation (21, 32).

An important finding of the present study is that both manual and automated voxel-based methods outperformed the conventional formula-based approach in discriminating csPCa. Compared with formula-based volumetry, voxel-based measurements yielded significantly higher AUCs not only for prostate volume itself but also for PSAD, while automated PSAD showed clinical utility broadly comparable to that of manual PSAD. Although the absolute improvement in AUC was modest, it is likely clinically meaningful in the PSA gray zone, where biopsy decisions often remain uncertain. This interpretation is further supported by decision curve analysis, which showed greater net benefit for voxel-based PSAD across clinically relevant threshold probabilities. Nevertheless, approximately 35% of csPCa cases would still have been missed at the Youden-optimal voxel-based PSAD cutoffs. Therefore, PSAD should not be used as a standalone criterion for biopsy decisions but should be interpreted together with PI-RADS and other clinical predictors. Together, these findings suggest that more accurate volumetry can improve the practical value of PSAD for risk stratification and biopsy decision-making. More importantly, by linking segmentation-derived volumetric measurements to the clinically relevant endpoint of csPCa detection within the same PSA gray zone cohort, our study extends previous work focused primarily on prostate volume estimation or segmentation accuracy and highlights the translational potential of automated voxel-based prostate volumetry in routine practice (21, 31, 33, 34).

Several limitations should be acknowledged. First, this was a retrospective single-center study, which may limit generalizability. Second, although the segmentation model was trained on a public dataset and externally validated before application to our cohort, further validation in larger multicenter populations and across different MRI protocols is still required. Third, csPCa was defined on the basis of biopsy and/or prostatectomy pathology, and biopsy-based classification remains subject to sampling error. Fourth, the PI-RADS 3 subgroup analysis was exploratory and included only 21 patients with csPCa; therefore, these findings require validation in a larger independent cohort. In addition, we did not perform a dedicated reclassification analysis within a prespecified borderline PSAD range. Fifth, we did not separately segment the peripheral zone and transition zone or calculate zone-specific PSA density. Given that prostate cancer arises from different zonal anatomies and that the diagnostic value of PSA density may vary between the peripheral and transition zones, this may have limited the ability of our analysis to capture more nuanced associations between zonal volume-adjusted PSA metrics and csPCa detection. Finally, the incremental multivariable models were developed and evaluated in the same single-center cohort and lacked external validation; therefore, their generalizability and calibration require further investigation.

## Conclusion

In conclusion, the method used for prostate volume estimation has a measurable impact on PSAD and its ability to detect csPCa in patients within the PSA gray zone. Formula-based volumetry tends to overestimate prostate volume and may reduce the discriminatory performance and clinical utility of PSAD. In contrast, voxel-based volume estimation, particularly when derived from automated segmentation, provides measurements that are closer to manual volumetry and yields improved performance for csPCa detection. These findings support the clinical utility of automated voxel-based prostate volumetry as a practical approach for optimizing PSAD-based risk stratification in patients with PSA levels of 4–10 ng/mL.

## Data Availability

The raw data supporting the conclusions of this article will be made available by the authors, without undue reservation.
