# Supplementary material for: Automated voxel-based prostate volumetry improves PSA density–based detection of clinically significant prostate cancer in the PSA gray zone: comparison with manual segmentation and the ellipsoid formula
Source: Front Digit Health. 2026 Oct 7;8:1897681. doi: 10.3389/fdgth.2026.1897681 (PMC13685902; doi:10.3389/fdgth.2026.1897681)
Supplement: Supplementary file 1 [file Table1.docx]

**Table S1** Diagnostic performance and biopsy-related outcomes of different PSAD methods in patients with PI-RADS 3 findings

| **PSAD method** | **Cutoff** | **Sensitivity** | **Specificity** | **PPV** | **NPV** | **Missed csPCa** | **Potential**  **biopsy avoidance** | **csPCa among**  **avoided biopsies** |
| --- | --- | --- | --- | --- | --- | --- | --- | --- |
| Formula-derived | 0.159 | 57.1% | 78.3% | 37.5% | 88.9% | 9/21 (42.9%) | 81/113 (71.7%) | 9/81 (11.1%) |
| Manual voxel-based | 0.170 | 71.4% | 71.7% | 36.6% | 91.7% | 6/21 (28.6%) | 72/113 (63.7%) | 6/72 (8.3%) |
| Automated voxel-based | 0.168 | 66.7% | 73.9% | 36.8% | 90.7% | 7/21 (33.3%) | 75/113 (66.4%) | 7/75 (9.3%) |

**Abbreviations**: csPCa, clinically significant prostate cancer; NPV, negative predictive value; PPV, positive predictive value; PSAD, prostate-specific antigen density.
